# Safety and Feasibility of Performing Fenestrated Endovascular Abdominal Aneurysm Repair Using a Portable C-arm Without Fusion Technology: A Single-Center Experience

**DOI:** 10.7759/cureus.7739

**Published:** 2020-04-20

**Authors:** Amandeep Juneja, Saqib Zia, Marco H Ayad, Kuldeep Singh, Jonathan Dietch, Jonathan Schor

**Affiliations:** 1 Surgery, Northwell Health, New York, USA; 2 Vascular Surgery, Staten Island University Hospital, Staten Island, USA; 3 Surgery, Staten Island University Hospital, Staten Island, USA

**Keywords:** endovascular, aortic aneurysm, radiation, c-arm, hybrid

## Abstract

Objective

Most centers performing fenestrated endovascular aneurysm repair (F-EVAR) use hybrid rooms with fusion technology for mapping. We present our experience of successfully performing F-EVAR using C-arm without fusion technology.

Methods

During the period of January 2016 to October 2018, data were collected from a prospectively maintained F-EVAR database at our tertiary care institute. The primary endpoint was technical success, and the secondary outcomes measured were short- and midterm clinical success (both defined by the Society for Vascular Surgery reporting standards), blood loss, radiation dose, operative time, postoperative endoleaks, aneurysm rupture, endograft patency, and complications.

Results

We performed 11 F-EVARs during the study period in five (45.5%) males and six (54.5%) females, with a mean age of 75+8 years. All procedures were performed under general anesthesia using OEC 9900 Elite Mobile C-arm (GE Healthcare, Chicago, IL, USA) without the use of fusion technology. Three patients had planned preoperative open procedures for access due to prior cutdown or bypass. Technical success was achieved in all 11 (100%) cases. The mean length of stay was 5+2 days, and the mean follow-up was 7.5+6.5 months. The mean procedure time was 301+167 minutes, and the mean blood loss was 361+233 mL. Mean fluoroscopy time was 72+31 minutes, and the mean radiation exposure time was 2,160+930 mGy. No patients required intraoperative transfusion. Thirty-day (short term) clinical success was achieved in 10 (90.0%), cases whereas six-month (midterm) clinical success was achieved in 7 (77.7%) patients. Branch vessel patency was 11 (100%) at 30 days and 9 (81.8%) at six months, and primary endograft patency was 100% (11) at six months. We had no perioperative mortality or major adverse cardiac event at 30 days. Thirty-day postoperative morbidity included readmission for pulmonary edema from cardiac failure in one patient. Two patients had clinically insignificant silent cardiac enzyme elevation. Three patients had re-interventions performed during the mean follow-up period. Two patients developed renal stent thrombosis resulting in renal insufficiency, which is defined as an increase in creatinine concentration ≥0.5 mg/dL, without the need for dialysis. One type II endoleak was identified postoperatively that required trans-lumbar embolization. No type I or III endoleaks were identified during the study period. Asymptomatic common femoral artery thrombosis was seen on follow-up imaging in one patient.

Conclusions

We conclude that F-EVAR can be safely performed using C-arm without the use of fusion technology. Its utility can be expanded to centers with appropriate skill set but no hybrid technology

## Introduction

Abdominal aortic aneurysm (AAA) is the most common type of aneurysm of the aorta. Current guidelines for screening in the United States of patients who are at increased risk of developing AAA has led to an increase in early diagnosis of asymptomatic AAA patients and reduction in aneurysm related mortality [1]. Advanced endovascular technology has allowed the repair of complex pararenal and juxtarenal aneurysm using endograft with fenestrations and physician-modified grafts. Most high-volume centers performing complex aneurysm repair use cone-beam computed tomography (CBCT) with fusion technology and fixed fluoroscopy units in a hybrid operating room setting. Limited data exist on complex aortic aneurysm repair using mobile C-arm without fusion technology. We performed pararenal/juxtarenal AAA repair using C-arm without fusion technology. We present the results from our case series of patients in the next section.

## Materials and methods

During the period of January 2016 to October 2018, data were collected from a prospectively maintained fenestrated endovascular aneurysm repair (F-EVAR) database at a tertiary care academic medical center. The primary endpoint was technical success defined as successful introduction and deployment of the device in the absence of surgical conversion or mortality, type I or III endoleaks, or graft limb obstruction. Secondary outcomes measured were short (30-day) and midterm (six-month) clinical success, blood loss, radiation dose, operative time, postoperative endoleaks, aneurysm rupture, endograft patency, and complications. Clinical success is defined by the Society of Vascular Surgery as successful deployment of the endovascular device at the intended location without death as a result of aneurysm-related treatment, type I or III endoleak, graft infection or thrombosis, aneurysm expansion (diameter ≥5 mm, or volume ≥5%), aneurysm rupture, or conversion to open repair. The presence of graft dilatation of 20% or more by diameter, graft migration, or a failure of device integrity classifies a case as a clinical failure [2].

The demographic data of the patients were collected, including age, gender, comorbidities such as diabetes, coronary artery disease, chronic obstructive pulmonary disease, end-stage renal disease, hypertension, and dyslipidemia, cerebrovascular accidents, and smoking history. All patients underwent FEVAR using Zenith® Fenestrated AAA Endovascular Graft Distal Bifurcated Body Grafts (ZFen, Cook Medical, Bloomington, IN, USA).

Perioperative data consisting of procedure time, blood loss, radiation doses, and complications were recorded. All patients were monitored in the surgical intensive care unit for 24 hours after the procedure and discharged if they have no postoperative complications. They were scheduled for the first postoperative office visit within four weeks after discharge. A postoperative computed tomography angiography (CTA) was obtained within four weeks to study the aneurysm. A second and third postoperative visit was scheduled at six-month intervals, and the aneurysm was studied using an ultrasound study at those follow-up visits. If no endoleak was noted, then follow-up was performed by ultrasound alone. If there was ultrasound evidence of endoleak or if graft failure was identified, then additional CTA evaluation was performed.

All continuous variables are reported as a mean ± standard deviation, and all categorical variables are reported as frequencies and percentages. The statistical analysis was performed using Statistical Package for the Social Sciences (SPSS), Version 24.0 (IBM Corp., Armonk, NY, USA).

## Results

Eleven F-EVAR procedures were performed during the study period: five (45.5%) in males and six (54.5%) in females, with a mean age of 75 + 8 years (Table 1).

**Table 1:** Demographics and Patient Characteristics CAD, coronary artery disease; ESRD, end-stage renal disease; CVA, cerebrovascular disease; PVD, peripheral vascular disease

Category	Frequency (n=11)	Percentages (%)
Males	5	45.5
Females	7	63.6
CAD	5	45.5
Hypertension	8	72.7
Diabetes	2	18.2
Dyslipidemia	6	54.5
ESRD	1	9.1
CVA	1	9.1
PVD	2	18.2
Tobacco Use	3	27.3

All procedures were performed under general anesthesia using OEC 9900 Elite Mobile C-arm (GE Healthcare, Chicago, IL, USA) without the use of fusion technology. Three patients required pre-operative bilateral iliofemoral bypasses due to inadequate iliac access for device delivery. These procedures were performed within four to six weeks of the scheduled F-EVAR procedure. One patient required a unilateral iliofemoral bypass performed as a single-stage procedure with the F-EVAR.

F-EVAR technical success was achieved in all 11 (100%) cases. The mean length of stay was 5+2 days, and the mean follow-up was 7.5+6.5 months. The mean procedure time was 301+167 minutes, and the mean blood loss was 361+233 mL. The mean fluoroscopy time was 72+31 minutes, and the mean radiation exposure time was 2160+930 mGy/cm^2^ (Table 2).

**Table 2:** Operative Details SD, standard deviation; BMI, body mass index; LOS, length of stay

Operative Details	Mean	SD
Age (year)	75	8.2
BMI	28	4.7
Aneurysm Diameter (cm)	6	1.1
Procedure Duration (minutes)	317	141.0
Blood Loss (mL)	355	241.0
Contrast (mL)	152	58.0
Fluoroscopy Time (minutes)	72	31.0
Radiation Exposure (mGy)	2160	930.0
Mean Follow-up (mo)	8	6.5
LOS (days)	5	2.0

No patients required intraoperative transfusion. Short-term (30-day) clinical success was achieved in 10 (90%) cases, whereas midterm (six-month) clinical success was achieved in 7 (77.7%) patients. The primary endograft patency (no intervention required to maintain patency) was 100% (11) at six months, and branch vessel patency was 11 (100%) at 30 days and 9 (81.8%) six months (Table 3).

**Table 3:** Operative Outcomes MACE, major adverse cardiac events

Category	Frequency (n=11)	Percentage (%)
Planned Percutaneous Access	8	72.7
Planned Open Access	3	27.3
Technical Success	11	100.0
Short-Term Clinical Success (30 days)	10	90.9
Midterm Clinical Success (six months)	7	77.7
Postoperative Endoleak	1	9.1
Short-Term Endograft Patency (30 days)	11	100.0
Midterm Endograft Patency (six months)	9	81.8
Systemic Complication	4	36.4
Local Complication	1	9.1
30-Day MACE	1	9.1

The total number of visceral vessels stented was 22 (21 renal arteries and 1 superior mesentery artery). Two patients developed renal stent thrombosis resulting in renal insufficiency, which is defined as an increase in creatinine concentration of ≥0.5 mg/dL, without the need for dialysis. Renal stent patency was successfully restored in one patient with thrombolysis. We had no perioperative mortality or major adverse cardiac event (MACE) at 30 days. Thirty-day postoperative morbidity included readmission for pulmonary edema from cardiac failure in one patient. Two patients had clinically insignificant silent cardiac enzyme elevation. Re-interventions were performed in three patients during the mean follow-up period, two for the renal artery stent thrombosis and one for type II endoleak that was identified postoperatively and required translumbar embolization, with successful resolution of the type II endoleak. No type I or III endoleaks were identified during the study period. Asymptomatic common femoral artery thrombosis was seen on follow-up imaging in one patient, and no intervention was performed for this.

## Discussion

The availability of fenestrated endografts has allowed the treatment of juxtarenal and pararenal abdominal aneurysms using endovascular techniques. Most high-volume centers performing these procedures use hybrid operating rooms with fusion technology. Our study looks at the feasibility and outcomes of performing these procedures in a standard operating room with mobile C-arm technology. The standard instruction for use (IFU) of infrarenal abdominal endovascular aortic repair (EVAR) device requires a minimum aneurysm neck length of 10-15mm between the renal artery and the proximal aspect of the graft. The minimum neck length is required to safeguard the renal arteries from occlusion by the proximal aspect of the graft while providing a long enough proximal landing zone for the graft. Although this criterion changes as more grafts become available, approximately 10% of patients with AAAs that are pararenal or juxtarenal do not qualify for standard EVAR due to unfavorable anatomy [3]. Prior to fenestrated technology, there was off-label use of renal stents through “snorkels” or “chimney” procedures in these patients with limited data and durability [4]. The availability of F-EVAR technology allowed many of these patients to be treated with a more stable and durable solution.

The novel distinguishing feature of the fenestrated endograft is its ability to envelop branches of the aorta without obstructing blood flow to these vessels. This is due to its fenestrations, or holes that are consistent with the position of the aortic branches and allow blood flow into these vessels while excluding flow into the aneurysm. These grafts are custom-designed and manufactured based on precise measurements using three-dimensional (3D) reconstructions of thin-slice CTA. The emergence of fenestrated grafts allows endovascular repair of juxtarenal and pararenal aortic aneurysms, which would otherwise require open repair [5].

Precise graft placement and complete exclusion of the aneurysm sac is the key to successful endovascular repair. CBCT and fusion technology allow better visualization of the 3D aortic sac and its branches which allows precise graft placement at lower radiation doses [6,7].

There are clear advantages of using fusion imaging, which allows cannulation of visceral and renal branches without using angiography using the fusion map. This lowers radiation exposure as well as contrast dose used [6,7]. Standard infrarenal EVAR using a mobile C-arm has shown to increase radiation exposure to both the patient and the provider. This fact is even more pronounced for complex EVARs. Custom manufactured devices such as the ZFen graft have shown a lower radiation dose exposure to the provider and the patient than other complex off-label repairs. This is likely due to the modeling of the graft based on 3D CT imaging of the aneurysm anatomy [8]. F-EVAR has been successfully performed using C-arm but two-dimensional imaging may not be adequate for all cases [8,9]. Mobile C-arm technology is cheaper, is more easily accessible, and has been used in select cases for F-EVAR. While CBCT and fusion technology have replaced conventional fluoroscopy in many institutions, there are still many facilities that lack this technology.

Radiation exposure to the patient and provider is always a concern when using fluoroscopy for cases. It is even more pronounced during longer and more complex cases. Studies have shown lower radiation exposure when using CBCT with fusion technology [10,11]. We observed fluoroscopy time and radiation exposure time of 72+31 minutes and 2,160+930 mGy/cm^2^, respectively, compared with 41+11 minutes and 1,380+530 mGy/cm^2^, respectively, in a study by Mcnally et al. using only 3D fusion computed tomography (CT) [12]. There are techniques to minimize radiation exposure using low-dose settings and pulse modes, which has shown lower exposures in standard EVAR. The same holds true for complex EVAR. Similarly, procedure duration and contrast use are longer when using C-arms without fusion technology [11]. Although alternatives to intravenous contrast are available, unfortunately there is no way to reduce procedure duration without using fusion technology and fixed equipment [12,13]. 

In terms of technical and clinical success and complications (endograft, branch vessels, systemic and local), there is no difference in our series compared with literature published by centers using CBCT and fusion technology [8,14-17].

Our study was limited by the number of patients and low power. This is the nature of a case series in a single institution. Comparing data for fenestrated/branched EVAR is difficult since the complexity of the cases is difficult to match. We did not perform a randomized control trial to study the differences in outcomes since we do not have access to CBCT and fusion technology.

## Conclusions

We conclude that F-EVAR can be safely performed using C-arm without the use of fusion technology. Its utility can be expanded to centers with appropriate endovascular experience but no hybrid technology. Although the use of hybrid rooms with fusion technology has some benefits, good outcomes can be achieved with the use of a mobile C-arm and no fusion technology. Despite the shortcomings of using a mobile C-Arm, complex F-EVAR and complex cases can have similar outcomes as those in a fixed and hybrid operating room.
